# Histopathological Features of Vanishing Testes in 332 Boys: What Is Its Significance? A Retrospective Study From a Tertiary Hospital

**DOI:** 10.3389/fped.2022.834083

**Published:** 2022-04-01

**Authors:** Lei Gao, Daxing Tang, Weizhong Gu

**Affiliations:** ^1^Department of Urology, National Clinical Research Center for Child Health, The Children's Hospital, Zhejiang University School of Medicine, Hangzhou, China; ^2^Department of Pathology, National Clinical Research Center for Child Health, The Children's Hospital, Zhejiang University School of Medicine, Hangzhou, China

**Keywords:** undescended testis, cryptorchidism, non-palpable testis, vanishing testis, pathology

## Abstract

The purpose of this study is to analyze the histopathological features of resected testicular remnant specimens, ascertain the incidence of the presence of either germ cells (GCs) or seminiferous tubules (SNTs), and assess whether surgical excision of the remnant is necessary. A total of 332 boys with vanishing testis underwent surgical removal of unilateral testicular remnants, with age 7–164 months (median age 25 months). Among the total 332 cases, 212 (63.8%) were younger than 36 months and 143 (66.5%) were found to have hypertrophied contralateral testes larger than 1.6 cm in longitudinal diameter under sonography. SNTs were only present in 21 (6.3%) cases and GCs were present in 7 (2.1%) cases. Compared to the review studies, the very low incidence of SNTs and GCs in which implies extremely low chances of potential malignancy. We propose that surgical removal of vanishing testis remnants in an inguinal or scrotal position may not be necessary.

## Introduction

Cryptorchidism or undescended testis (UDT) is reported in 1–4.6% of full-term and 1.1–45.3% of premature male infants (1). Approximately 20% of UDT cases are found to be clinically non-palpable (2). In some cases, there is a small abnormal testicular remnant, which is referred to as vanishing testis (3). Vanishing testis, which is often referred to as testicular regression syndrome in the recent medical literature and which accounts for 35–60% cases of non-palpable testis (NPT), is characterized by a rudimentary spermatic cord with absence of macroscopically identifiable testicular tissue (3–6). Usually, a small fibrotic nodule or nubbin is found at the end of this spermatic cord. Fibrosis, dystrophic calcification, and hemosiderin deposition in association with identifiable testicular or paratesticular structures are common features of vanishing testis in histopathology (7, 8). These raise the questions of whether vanishing testis was the result of testicular torsion or a vessel accident, or was just a hypoplasia during the fetal phase. The optimal management of the testicular remnant is controversial, depending on the presence of seminiferous tubules (SNTs) and/or germ cells (GCs) that indicate a potential for malignancy if not excised (3, 5, 9–11). In this study, we conducted a retrospective review with a large sample of vanishing testis, analyzed the clinical characteristics of patients with vanishing and histopathological features of resected testicular remnant specimens, ascertained the incidence of the presence of GCs and SNTs, and assessed whether surgical excision of the remnant is necessary.

## Materials and Methods

**Figure 1:**
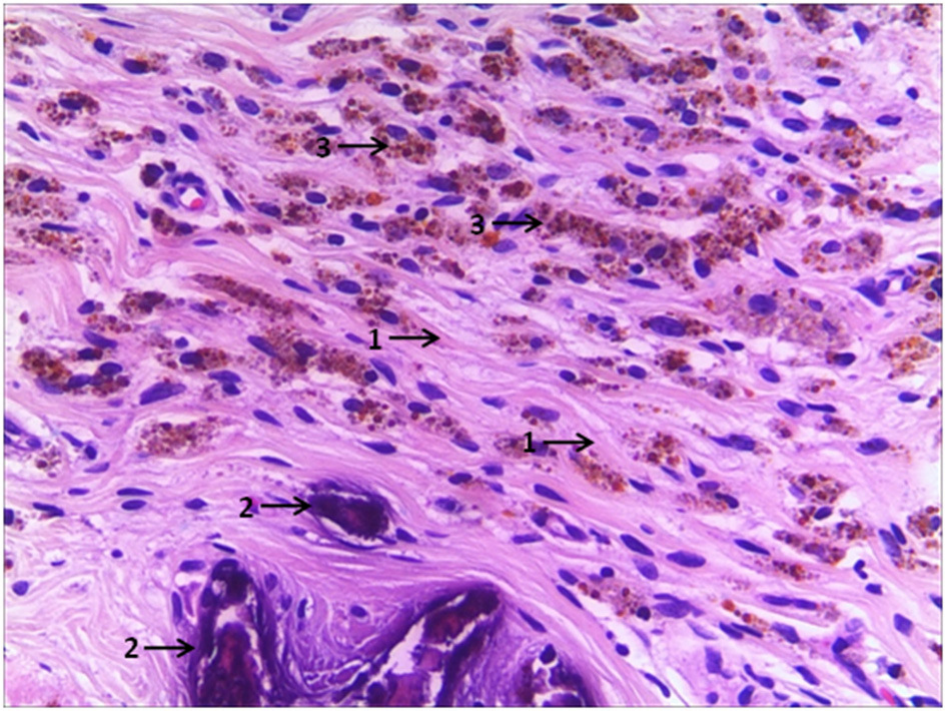
A case of vanishing testis showing fibrosis (arrow 1), dystrophic calcifications (arrow 2), and hemosiderin deposits (arrow 3) in H&E stain. Images under ×200 visual field.

A retrospective review was performed for pediatric patients identified as unilateral testicular remnant consistent with vanishing testis after surgical excision between July 2008 and November 2018. Data were collected from surgical management details from both clinical notes and operative records. Pre-operative inguinal or scrotal ultrasound data in detecting non-ppalpable and contralateral testis were collected, including the location of the identified remnant in the affected side and the size of the contralateral testis. Histological findings including fibrosis, calcification, hemosiderin deposition (Figure 1), remnants of testicular or paratesticular tissue and spermatic cord were noted. Patients that had undergone excision of an atrophied testicle following a previous orchidopexy, testicular torsion or incarcerated inguinal hernia, and patients with bilateral testicular remnants were excluded from the study.

In our institution, all patients with clinical NPT undergo physical examination under anesthetic, and patients whose testes are still non-palpable proceed to a laparoscopy. During the laparoscopy, if no intra-abdominal testis is found and the spermatic cord enters into a closed internal ring, the inguinal or scrotal region is explored. A small fibrotic nodule or nubbin with no macroscopically normal testicular tissue identified is found at the end of the spermatic cord, and any testicular remnant is excised then. If a macroscopically abnormal intra-abdominal testicular remnant is revealed at laparoscopy, it is excised subsequently. Other patients with palpable nubbin in the inguinal or scrotal region undergo a direct inguinal or scrotal exploration instead of laparoscopy.

Histologic confirmation of testicular or paratesticular tissue required the presence of at least one of the following: SNTs, GCs, Sertoli cells, Leydig cells (testicular tissue) and vas deferens, or epididymal structures (paratesticular tissue). Identification of specimens with SNTs and viable GCs was confirmed by an expert pathologist. Hematoxylin and eosins (H&E), monoclonal mouse antibody Oct3/4 (clone N1NK, DAKO), monoclonal mouse antibody Sal-like protein 4 (Sall4) (clone EE30, Santa Cruz) stains were used to document the presence of GCs, while H&E, monoclonal mouse anti-androgen receptor (AR) (clone AR441, DAKO) and polyclonal goat anti-anti-müllerian hormone (AMH) (MIS C-20, sc-6886, Santa Cruz) stains were used to identify the presence of SNTs, which consist of Sertoli cells and Leydig cells.

Results were presented as median (range) or absolute numbers (percentages), as appropriate. SPSS for Windows v20 (SPSS, Chicago, IL) was used for analysis. Data analysis included Fisher's exact test, and a *P* < 0.05 was regarded as statistically significant.

## Result

A total of 332 cases of testicular remnants are summarized. Median (range) age at surgery was 25 (7–164) months. Among these cases, the remnants of 258 (77.7%) cases were left sided (*P* < 0.05). 41 (12.5%) nubbins were palpated in the inguinal or scrotal region. Within the 212 (63.8%) cases of patients younger than 36 months, 143 (66.5%) had hypertrophied contralateral testes and the sizes were larger than 1.6 cm in longitudinal diameter measured by sonography. 291 (87.5%) of the 332 patients underwent laparoscopic exploration; whereas the remaining 41 (12.5%) patients underwent direct inguinal or scrotal exploration initially.

Routine histologic studies showed fibrosis in 275 (82.8%), dystrophic calcification in 99 (29.8%), hemosiderin deposition in 39 (11.7%), vas deferens in 187 (56.3%) and epididymis in 111 (33.4%) cases. SNTs were present in 21 (6.3%) cases. GCs were available only in 7 (2.1%) cases with ages ranging from 10 to 126 months, including one intra-abdominal case (Table 1). Comparison of these 4-year age subgroups of patients revealed no significant differences with the incidence of SNTs or GCs *(P* > 0.05). A summary of the four subgroups and a overall analysis is presented (Table 2). According to our long-term follow-up observation for these vanishing testes patients aged 7–164 months with excision, no occurrence of malignancy has been found until now.

**Table 1:** Summary of the histological characteristics for vanishing testis specimens (*n* = 332).

**Histological features**	**Total number**	**%**
Fibrosis	275	82.8%
Dystrophiccalcification	99	29.8%
Haemosiderin deposition	39	11.7%
Vas deferens	187	56.3%
Epididymis	111	33.4%
SNTs	21	6.3%
	(9 in inguinal, 11 in scrotum and 1 in abdomen)	
GCs	7	2.1%
	(5 in inguinal,1 in scrotum and abdomen)	

**Table 2:** Summury of the four subgroups.

	**7–12 months *n* (%)**	**12–36 months *n* (%)**	**36–108 months *n* (%)**	**108–216 months *n* (%)**	***P*-value**	**Overall**
Patients	33	179	97	23	-	332
SNTs	2 (6.1%)	14 (7.8%)	2 (2.1%)	3 (13.0%)	0.085	21 (6.3%)
GCs	1 (3.0%)	3 (1.7%)	2 (2.1%)	1 (4.3%)	0.554	7 (2.1%)

**Figure 2:**
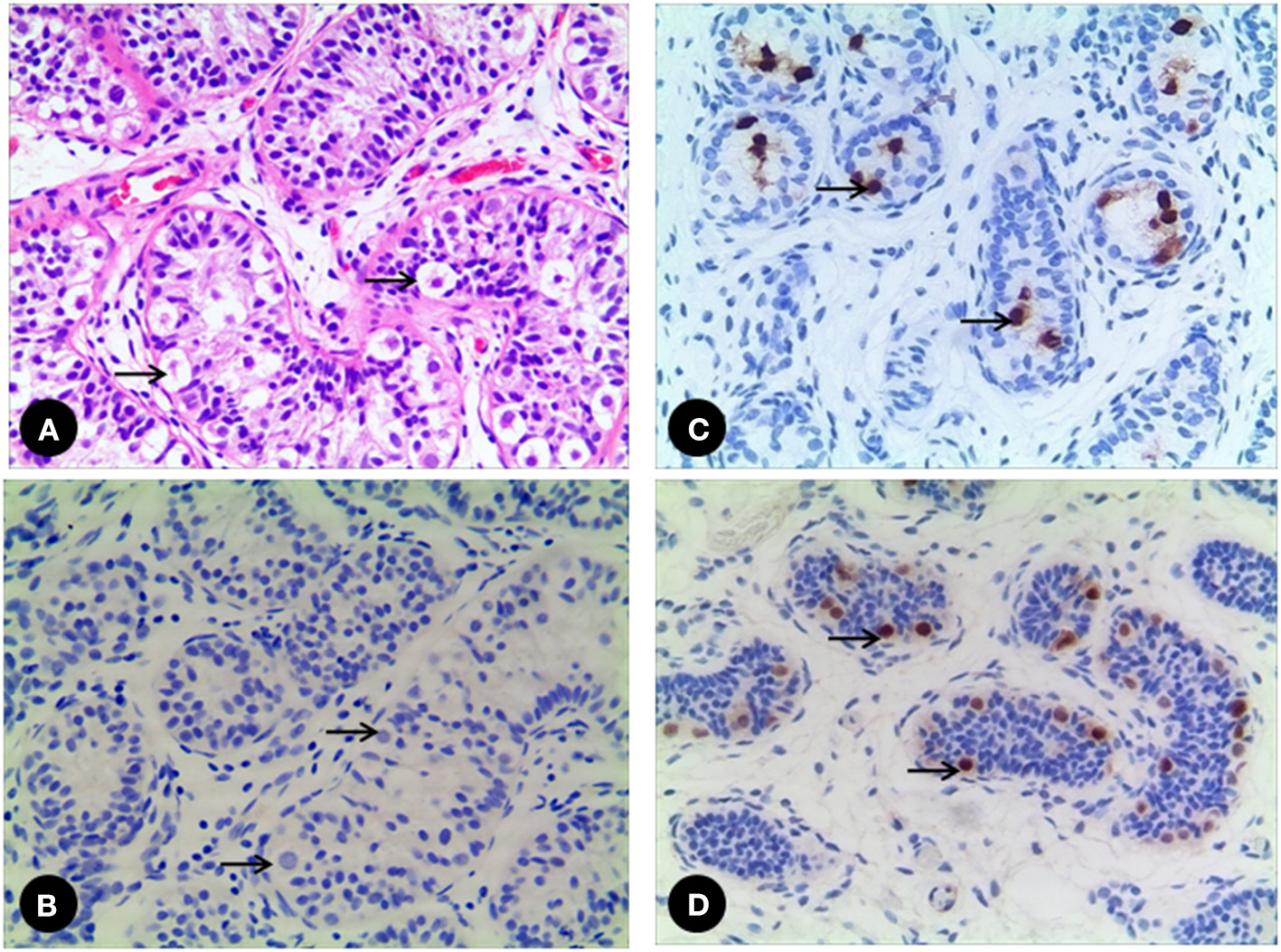
**(A)** A 52 month-old vanishing testis patient. The GCs (arrow) are easily identified in H&E stain, **(B)** while the GCs (arrow) are negative to Oct3/4. **(C)** A 17 month-old vanishing testis patient showing the GCs (arrow) with nuclear positive for Oct3/4. **(D)** Another 28 month-old vanishing testis patient showing the GCs (arrow) with nuclear positive for Sall4. Images under ×200 visual field.

**Figure 3:**
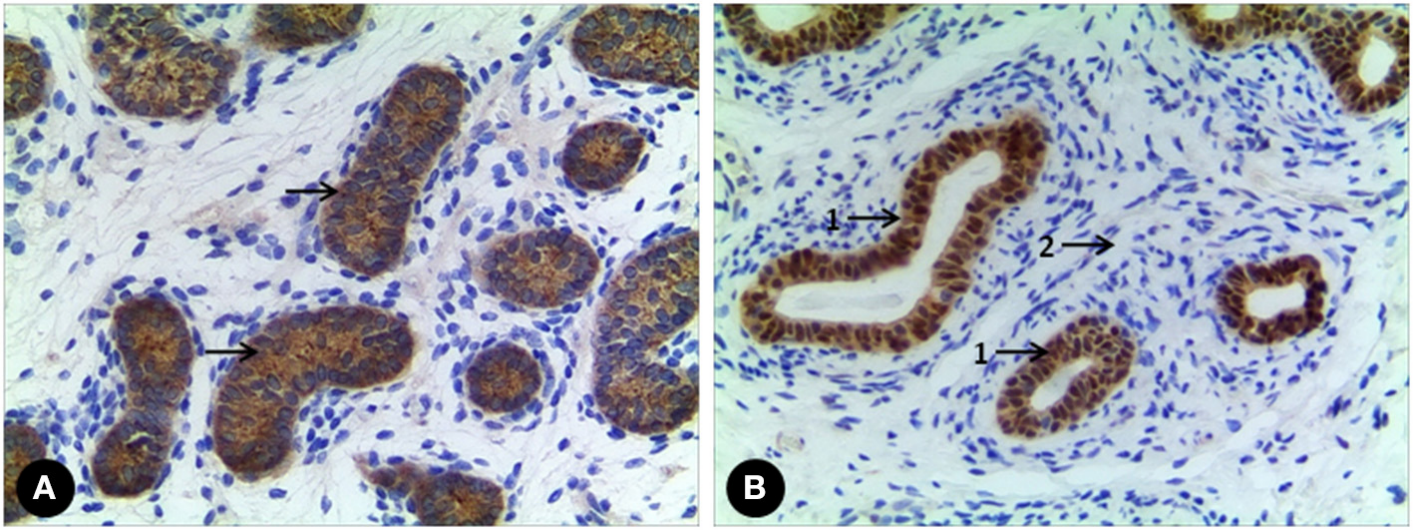
**(A)** A case of vanishing testis showing Sertoli cell population with immunohistochemical cytoplasmic positivity for anti-AMH (arrow) in SNTs. **(B)** Another one showing Sertoli cell population with immunohistochemical nuclear positivity for anti-AR (arrow 1) in SNTs, but no Leydig cell with immunohistochemical positivity for anti-AR in interstitial space (arrow 2). Images under ×100 visual field.

All 7 GCs patients were positive for Sall4, while only 2 cases were positive for Oct3/4 (Figure 2). No recognizable Leydig cells positive for anti-AR were present in the stroma surrounding the SNTs, which are positive for anti-AMH and anti-AR (Figure 3). The immunohistochemical findings of vanishing testis specimens with GCs are summarized (Table 3).

**Table 3:** The immunohistochemical findings of vanishing testis specimens with GCs.

**Case NO**.	**Age (M)**	**Laterality**	**Location**	Anti- AMH^*^	Anti- AR^*^	Anti- AR^**^	Oct3/4^***^	Sall4^***^
1	10	Left	Inguinal	**+**	**+**	**-**	**+**	**+**
2	15	Right	Inguinal	**+**	**+**	**-**	**-**	**+**
3	17	Right	Abdomen	**+**	**+**	**-**	**+**	**+**
4	28	Left	Inguinal	**+**	**+**	**-**	**-**	**+**
5	43	Right	Inguinal	**+**	**+**	**-**	**-**	**+**
6	52	Left	Scrotum	**+**	**+**	**-**	**-**	**+**
7	126	Right	Inguinal	**+**	**+**	**-**	**-**	**+**

* *Localizing Sertoli cells*,

** *Localizing the stroma (Ledig cells)*,

*** *Localizing the GCs*.

## Discussion

Vanishing testis, which was coined by Abeyaratne et al. (12), refers to the finding of blind ending spermatic vessels and vas deferens during the exploration of UDT (12). From 1980 to 2016, a total of 1,455 vanishing testis specimens from pediatric patients were reported in 29 studies, which contained data that were relevant to the incidence of GCs and/or SNTs in resected vanishing testis specimens (13). To the best of our knowledge, our study of 332 cases includes the largest individual sample of vanishing testis in the literature. Owing to a lack of consensus, there are various definitions of vanishing testis (4, 5, 9, 14). In this study, we used a widely accepted definition: a congenital condition in which no macroscopically normal testicular tissue can be identified following exploration for a clinically NPT (14). This definition includes the presence of a testicular remnant, nodule or strand of testicular or paratesticular tissue at the end of the spermatic cord. Other definitions include the presence of a hypoplastic spermatic vessels and vas deferens entering a closed ring or blind-ending spermatic vessels visualized in the retroperitoneum, as revealed on laparoscopy (10). Although the etiology, pathogenesis, and pathophysiology of vanishing testis have not been fully established, recent studies strongly support a vascular accident and antenatal torsion theory rather than an endocrinopathy (6). This hypothesis was supported in our study by the frequent presence of fibrosis, dystrophic calcification, and hemosiderin deposition in the vanishing testis specimens. These pathological features are similar to the results of our previous animal experiment (15). Furthermore, as stated in our study and in other reports (5), testicular remnants are found more commonly on the left. Kinking of left testicular vein because of its anatomical relationship to the left renal vein with an unusually mobile kidney is presumed to be a secondary cause of vascular obstruction (16).

Enlargement of the contralateral descended testis is considered to be the most helpful clinical sign of vanishing testis (17, 18). This finding can provide useful information for parental counseling and for clinical treatment planning. In 1969 Laron and Zilka first observed the phenomenon of contralateral compensatory testicular hypertrophy in monorchism in humans, after this phenomenon had been studied extensively in unilaterally castrated animals (19). Two decades later, Koff was the first to measure the lengths and volumes of contralateral descended testes in 37 patients with NPT, and 12 monorchism were found with mean contralateral testicular length larger than 2 cm, compared to the size of 1.6 cm or less for viable testes. An intermediate group with atrophic ipsilateral testes had contralateral measurements of 1.6–2 cm (20). Similarly, Snodgrass reported that monorchism was strongly associated with a contralateral descended testicular length of 1.8 cm or greater (18). In our study, 143 of 212 vanishing testis patients younger than 36 months, were found to have hypertrophied contralateral testis larger than 1.6 cm by ultrasound. Our findings are consistent with the existing literature.

There is controversy regarding the optimal management of vanishing testis patients. Some authors recommend surgical exploration, via either laparoscopic or inguinal or scrotal approach, whereas other authors believe that these procedures are unnecessary (3, 9). Laparoscopy, which was introduced by Cortesi et al. (21), has been the most sensitive and specific modality to evaluate patients with NPT and has provided surgeons with an algorithm for surgical approach for almost 40 years (22). Nowadays most pediatric surgical centers advocate that a patient with NPT should undergo an examination under anesthetic and laparoscopy in order to exclude the presence of an intra-abdominal testicle. In our study, 291 (87.5%) patients, of whom no nubbin was identified in the inguinal or scrotal region by physical examination, underwent laparoscopic exploration initially. During laparoscopic exploration, a closed internal ring with an intra-abdominal blind-ending vessel or with hypoplastic spermatic vessels and vas deferens entering without an abdominal testis were observed except for one intra-abdominal testicular remnant. Inguinal or scrotal exploration followed and comfirmed the remnants, of which no macroscopically normal testicular tissue can be identified. These laporoscopic findings were always associated with the presence of an extra-abdominal nubbin in unilateral NPT (23). In our study the other 41 patients with nubbin detected in the inguinal or scrotal region underwent direct inguinal or scrotal exploration and remnant excision. This surgical approach was regarded as potentially definitive for the diagnosis and management of vanishing testis (18). Some authors have recommended a modified surgical procedure of a median raphe scrotal incision, which is made not only to remove the testicular remnants, but also to enable the inspection and/or fixing of a contralateral testis (9). In our experience, if we are going to excise the remnant, the surgical approach of initial inguinal or scrotal exploration in vanishing testis patients is recommended when the nubbin is clearly detected in the inguinal or scrotal region by physical examination, especially with compensatory enlargement of the contralateral descended testis.

Currently, the optimal management of the testicular remnant of vanishing testis is still under debate. Some advocate routine surgical exploration to remove the remnant, whereas others question its necessity (3–5, 7, 9–11, 24). These two opinions are based on the variance of SNTs (0–34%) and viable GCs (0–15.4%) elements within the remnants in the reported incidences and the subsequent risk of potential malignant degeneration (13). One concern with SNTs is the possible presence of GCs, although they may not be identified in histological analysis. In our study, Sall4, which is positive for GCs (spermatogonia of testis) (25), was used together with H&E stain to identify GCs with a positive rate of 100%. In contrast, the positive rate of using Oct3/4 is only 29%, which is positive for fetal GCs and for some GCs of the neonatal age and early infancy (26). This discrepancy could be attributed to the different immunohistochemical analysis practices in different studies. In our study, an individual pathologist performed the histological analysis; so this source of bias was minimized. In our study, the percentages of GCs and SNTs found in the specimens were somewhat lower compared to previous cohorts (13), there was significant difference indeed (GCs 2.1 vs. 5.3%, *p* = 0.03, SNTs 6.3 vs. 10.7%, *p* = 0.04). However, there was a large range of GCs (0–15.4%) and SNTs (0–34%) incidence in the previous cohorts studies, in which significant heterogeneity was observed. It is obvious that our data are also in the range, but it seems as if the credibility of our analysis results was higher.

To the best of our knowledge, it is well-known that there is increased future malignancy risk with congenital UDT with a standardized incidence ratio of 2.23 for younger children (<13 years old at operation) and 5.4 for adolescents (>13 years old at operation) (27). Clinically and histologically, germ cell testicular cancer are categorized by seminomatous and non-seminomatous tumors, and the latter may be composed of teratomas, choriocarcinoma, yolk-sac tumors, embryonal carcinomas, and mixed germ-cell tumors (27, 28). However, the etiology of vanishing testis differs from that of a congenital UDT, and the future malignancy risk also differs (13). Thus, far, the only reported case of intratubular germ cell neoplasia in a remnant testicle was in a 9-year-old boy; however, it was not supported immunohistochemically (24). In the present study, we found an overall incidence rate of 6.3% for SNTs and 2.1% for GCs, and there was no evidence that the incidence of SNTs or GCs increases with the age of patient. The indication for intervention is therefore not age-dependent, although smaller patient numbers in the older age groups limited this subset analysis. So far, no occurrence of malignancy has been found in long-time follow-up observation of these vanishing testis patients in our institution. Therefore, according to our study and data from the literature, the low incidence rate of viable tissue within the testicular nubbin, particularly the rare occurrence of GCs, does not support the necessity of surgical excision. Because all testicular tumors originate from solid cells (Sertoli cell, Leydig cell and germ cell) (15), if there is almost no solid cell is left in the testicular remnants, the risk of future malignant transformation should be minimal. Furthermore, most remnants in the inguinal or scrotal region can easily be detected and excised in cases of early malignant changes. However, intra-abdominal vanishing testis remnants may contain more elements and therefore may require excision (11), which was performed in one vanishing testis patient with presence of GCs and SNTs in our study. Some authors recommend an alternative surgical management wherein inguinal exploration is postponed until the implantation of testicular prosthesis, which is a cosmetic option for patients with unilateral or bilateral loss of testes. Under this surgical approach, testicular prosthesis implantation can be performed as a first inguinal operation and testicular remnants can be removed at the same time (4).

Our study included several limitations, such as a reliable comparison group of children who did not have remnants excised and their long-term follow-up, and further analysis of costs and risks of performing potentially unnecessary resection (i.e., operative time, wound pain, bleeding, infection, etc.). Further study to address these inadequacies would be valuable. Other limitations of our study were the small number of patients, especially older patients, and the inclusion of only pediatric patients ≤18 years old. The vanishing testis hypothetical future malignancy risk is lifelong, although there are no published findings which prove that vanishing testis is linked to malignancy during adulthood. A multi-center study with lifelong follow-up should be carried out to justify the surgical management.

## Conclusion

In this study, we propose that surgical removal of vanishing testis remnants in an inguinal or scrotal position may not be necessary owing to the very low incidence of SNTs and GCs in resected testicular remnant specimens. However, excision of intra-abdominal vanishing testis remnants is still necessary because intra-abdominal remnants may contain more elements and are difficult to monitor.

## Data Availability Statement

The original contributions presented in the study are included in the article/supplementary material, further inquiries can be directed to the corresponding author.

## Ethics Statement

The studies involving human participants were reviewed and approved by the Ethics Board of the above Hospital. Written informed consent to participate in this study was provided by the participants' legal guardian/next of kin.

## Author Contributions

LG: guarantor of integrity of the entire study, study concepts, study design, definition of intellectual content, literature research, clinical studies, experimental studies, data acquisition, data analysis, statistical analysis, manuscript preparation, and manuscript editing. DT: guarantor of integrity of the entire study, study concepts, study design, definition of intellectual content, literature research, clinical studies, data analysis, manuscript preparation, manuscript editing, and manuscript review. WG: clinical studies, experimental studies, and data acquisition. All authors contributed to the article and approved the submitted version.

## Funding

This work was supported by funding from Major State Basic Research Development Program of China (Grant Number 2018YFC1002700).

## Conflict of Interest

The authors declare that the research was conducted in the absence of any commercial or financial relationships that could be construed as a potential conflict of interest.

## Publisher's Note

All claims expressed in this article are solely those of the authors and do not necessarily represent those of their affiliated organizations, or those of the publisher, the editors and the reviewers. Any product that may be evaluated in this article, or claim that may be made by its manufacturer, is not guaranteed or endorsed by the publisher.
